# Identification of Essential Oils with Strong Activity against Stationary Phase *Borrelia burgdorferi*

**DOI:** 10.3390/antibiotics7040089

**Published:** 2018-10-16

**Authors:** Jie Feng, Wanliang Shi, Judith Miklossy, Genevieve M. Tauxe, Conor J. McMeniman, Ying Zhang

**Affiliations:** 1 Department of Molecular Microbiology and Immunology, Bloomberg School of Public Health, Johns Hopkins University, Baltimore, MD 21205, USA; jfeng16@jhu.edu (J.F.); wshi3@jhu.edu (W.S.); gtauxe1@jhu.edu (G.M.T.); cmcmeni1@jhu.edu (C.J.M.); 2 International Alzheimer Research Centre, Prevention Alzheimer International Foundation, Martigny-Croix CP 16 1921, Switzerland; judithmiklossy@bluewin.ch

**Keywords:** *Borrelia burgdorferi*, persisters, biofilm, antimicrobial activity, essential oils

## Abstract

Lyme disease is the most common vector borne-disease in the United States (US). While the majority of the Lyme disease patients can be cured with 2–4 weeks antibiotic treatment, about 10–20% of patients continue to suffer from persisting symptoms. While the cause of this condition is unclear, persistent infection was proposed as one possibility. It has recently been shown that *B. burgdorferi* develops dormant persisters in stationary phase cultures that are not killed by the current Lyme antibiotics, and there is interest in identifying novel drug candidates that more effectively kill such forms. We previously identified some highly active essential oils with excellent activity against biofilm and stationary phase *B. burgdorferi.* Here, we screened another 35 essential oils and found 10 essential oils (*Allium sativum* L. bulbs, *Pimenta officinalis* Lindl. berries, *Cuminum cyminum* L. seeds, *Cymbopogon martini* var. *motia* Bruno grass, *Commiphora myrrha* (T. Nees) Engl. resin, *Hedychium spicatum* Buch.-Ham. ex Sm. flowers, *Amyris balsamifera* L. wood, *Thymus vulgaris* L. leaves, *Litsea cubeba* (Lour.) Pers. fruits, *Eucalyptus citriodora* Hook. leaves) and the active component of cinnamon bark cinnamaldehyde (CA) at a low concentration of 0.1% have strong activity against stationary phase *B. burgdorferi*. At a lower concentration of 0.05%, essential oils of *Allium sativum* L. bulbs, *Pimenta officinalis* Lindl. berries, *Cymbopogon martini* var. *motia* Bruno grass and CA still exhibited strong activity against the stationary phase *B. burgdorferi*. CA also showed strong activity against replicating *B. burgdorferi*, with a MIC of 0.02% (or 0.2 μg/mL). In subculture studies, the top five essential oil hits *Allium sativum* L. bulbs, *Pimenta officinalis* Lindl. berries, *Commiphora myrrha* (T. Nees) Engl. resin, *Hedychium spicatum* Buch.-Ham. ex Sm. flowers, and *Litsea cubeba* (Lour.) Pers. fruits completely eradicated all *B. burgdorferi* stationary phase cells at 0.1%, while *Cymbopogon martini* var. *motia* Bruno grass, *Eucalyptus citriodora* Hook. leaves, *Amyris balsamifera* L. wood, *Cuminum cyminum* L. seeds, and *Thymus vulgaris* L. leaves failed to do so as shown by visible spirochetal growth after 21-day subculture. At concentration of 0.05%, only *Allium sativum* L. bulbs essential oil and CA sterilized the *B. burgdorferi* stationary phase culture, as shown by no regrowth during subculture, while *Pimenta officinalis* Lindl. berries, *Commiphora myrrha* (T. Nees) Engl. resin, *Hedychium spicatum* Buch.-Ham. ex Sm. flowers and *Litsea cubeba* (Lour.) Pers. fruits essential oils all had visible growth during subculture. Future studies are needed to determine if these highly active essential oils could eradicate persistent *B. burgdorferi* infection in vivo.

## 1. Introduction

Lyme disease, which is caused by the spirochetal organism *Borrelia burgdorferi,* is the most common vector borne-disease in the United States (US) with about 300,000 cases a year [1]. While the majority of the Lyme disease patients can be cured with the standard 2–4 weeks antibiotic monotherapy with doxycycline or amoxicillin or cefuroxime [2], about 36% of patients continue to suffer from persisting symptoms of fatigue, joint, or musculoskeletal pain, and neuropsychiatric symptoms, even six months after taking the standard antibiotic therapy [3]. These latter patients suffer from a poorly understood condition, called post-treatment Lyme disease (PTLDS) syndrome. While the cause for PTLDS is unclear and is likely multifactorial, the following factors may be involved: autoimmunity [4], host response to dead debris of *Borrelia* organism [5], tissue damage caused during the infection, and persistent infection. There have been various anecdotal reports demonstrating persistence of the organism despite standard antibiotic treatment [6,7,8]. For example, culture of *B. burgdorferi* bacteria from patients despite treatment has been reported as infrequent case reports [9]. In addition, in animal studies with mice, dogs and monkeys, it has been shown that the current Lyme antibiotic treatment with doxycycline, cefuroxime, or ceftriaxone is unable to completely eradicate the *Borrelia* organism, as detected by xenodiagnosis and PCR [6,7,8,10], but viable organism cannot be cultured in the conventional sense as in other persistent bacterial infections, like tuberculosis after treatment [11,12].

Recently, it has been demonstrated that *B. burgdorferi* can form various dormant non-growing persisters in stationary phase cultures that are tolerant or not killed by the current antibiotics that are used to treat Lyme disease [13,14,15,16]. Thus, while the current Lyme antibiotics are good at killing the growing *B. burgdorferi* they have poor activity against the non-growing persisters enriched in stationary phase culture [14,16,17]. Therefore, there is interest to identify drugs that are more active against the *B. burgdorferi* persisters than the current Lyme antibiotics. We used the stationary phase culture of *B. burgdorferi* as a persister model and performed high throughput screens and identified a range of drug candidates such as daptomycin, clofazimine, sulfa drugs, daunomycin, etc., which have strong activity against *B. burgdorferi* persisters. These persister active drugs act differently from the current Lyme antibiotics, as they seem to preferentially target the membrane. We found that the variant persister forms such as round bodies, microcolonies, and biofilms with increasing degree of persistence in vitro, cannot be killed by the current Lyme antibiotics or even persister drugs like daptomycin alone, and that they can only be killed by a combination of drugs that kill persisters and drugs that kill the growing forms [14]. These observations provide a possible explanation in support of persistent infection despite antibiotic treatment in vivo.

Although daptomycin has good anti-persister activity, it is expensive and is an intravenous drug and difficult to administer and adopt in clinical setting and it has limited penetration through blood brain barrier (BBB). Thus, there is interest to identify alternative drug candidates with high anti-persister activity. We recently screened a panel of 34 essential oils and found the top three candidates oregano oil and its active component carvacrol, cinnamon bark, and clove bud as having even better anti-persister activity than daptomycin at 40 μM [18]. To identify more essential oils with strong activity against *B. burgdorferi* persisters, in this study, we screened an additional 35 different essential oils and found 10 essential oils (garlic, allspice, cumin, palmarosa, myrrh, hydacheim, amyris, thyme white, *Litsea cubeba*, lemon eucalyptus) and the active component of cinnamon bark cinnamaldehyde as having strong activity in the stationary phase *B. burgdorferi* persister model. 

## 2. Materials and Methods

### 2.1. Organism and Culture Conditions

A low passaged strain *B. burgdorferi* B31 5A19 was kindly provided by Dr. Monica Embers [15]. Firstly, we prepared the *B. burgdorferi* B31 culture in BSK-H medium (HiMedia Laboratories, Mumbai, India), supplemented with 6% rabbit serum (Sigma-Aldrich, St. Louis, MO, USA) without antibiotics. After incubation for seven days in microaerophilic incubator (33 °C, 5% CO_2_), the *B. burgdorferi* culture went into stationary phase (~10^7^ spirochetes/mL), followed by evaluating potential anti-persister activity of essential oils in a 96-well plate (see below). 

### 2.2. Essential Oils and Drugs

We purchased a panel of essential oils (Plant Guru, Plainfield, NJ, USA) and cinnamaldehyde (CA) (Sigma-Aldrich, St. Louis, MO, USA). The essential oils from Plant Guru company are tested by third party laboratory using GC/MS, and the GC/MS report can be found on their website [19]. Dimethyl sulfoxide (DMSO)-soluble essential oils were prepared at 10% (*v/v*) in DMSO as stock solution, which was then added with seven-day old stationary phase cultures at ration of 1:50 to achieve 0.2% of essential oils in the mixture. The 0.2% essential oils were further diluted to the stationary phase culture to get the desired concentration for evaluating anti-borrelia activity. DMSO-insoluble essential oils were directly added to *B. burgdorferi* cultures, then vortexed to form aqueous suspension, followed by immediate transfer of essential oil aqueous suspension in serial dilutions to desired concentrations and then added to *B. burgdorferi* cultures. Doxycycline (Dox), cefuroxime (CefU), (Sigma-Aldrich, St. Louis, MO, USA), and daptomycin (Dap) (AK Scientific, Union City, CA, USA) were prepared at a concentration of 5 mg/mL in suitable solvents [20,21], then filter-sterilized by 0.2 μm filter and stored at −20 °C as stock solutions. 

### 2.3. Microscopy

Treated *B. burgdorferi* cell suspensions were checked with BZ-X710 All-in-One fluorescence microscope (KEYENCE, Itasca, IL, USA). The bacterial viability was evaluated by SYBR Green I/PI assay, which was performed by calculating the ratio of green/red fluorescence after dying to determine the ratio of live and dead cells, as described previously [16,22]. The residual cell viability reading was obtained by analyzing three representative images of the same bacterial cell suspension taken by fluorescence microscopy. To quantitatively determine the bacterial viability from microscope images, software of BZ-X Analyzer and Image Pro-Plus were employed to evaluate fluorescence intensity, as we described previously [14].

### 2.4. Evaluation of Essential Oils for Their Activities Against B. Burgdorferi Stationary Phase Cultures

To evaluate the possible activity of the essential oils against stationary phase *B. burgdorferi*, 10% DMSO-soluble essential oils or aqueous suspension of DMSO-insoluble essential oils were added to 100 µL of the seven-day old stationary phase *B. burgdorferi* culture in 96-well plate to obtain the desired concentrations. In the primary screen, each essential oil was assayed with final concentrations of 0.2% and 0.1% (*v/v*) in 96-well plates. Drugs of daptomycin, doxycycline, and cefuroxime were used as control with final concentration of 40 μM. The active hits were checked further with lower concentrations of 0.1% and 0.05%; all of the tests mentioned above were run in triplicate. All of the plates were sealed and incubated at 33 °C without shaking for seven days, and 5% CO_2_ were maintained in the incubator. 

### 2.5. Essential Oil and Drug Susceptibility Testing

The live and dead cells after seven-day treatment with essential oils or antibiotics were evaluated using the SYBR Green I/PI assay combined with fluorescence microscopy, as described [16,22]. Briefly, the ratio of live and dead cells was reflected by the ratio of green/red fluorescence, which was calculated through the regression equation and regression curve with least-square fitting analysis. 

To determine the Minimum inhibitory concentration (MIC) of cinnamaldehyde on growth of *B. burgdorferi*, the standard microdilution method was used and the growth inhibition was assessed by microscopy. 10% cinnamaldehyde DMSO stock was added to *B. burgdorferi* cultures (1 × 10^4^ spirochetes/mL) to get an initial suspension with 0.5% of cinnamaldehyde, and then a series of suspension was prepared by two-fold dilutions, with cinnamaldehyde concentrations ranging from 0.5% (=5 µg/mL) to 0.004% (=0.04 µg/mL). All of the experiments were carried out in triplicate. The *B. burgdorferi* cultures after treatment in 96-well microplate were incubated at 33 °C for seven days. Cell proliferation was assessed by the SYBR Green I/PI assay combined with BZ-X710 All-in-One fluorescence microscope.

### 2.6. Subculture Studies to Assess Viability of Essential Oil-Treated B. Burgdorferi Organisms

Essential oils or control drugs were added into 1 mL of seven-day old *B. burgdorferi* stationary phase culture in 1.5 mL Eppendorf tubes, incubated for seven days at 33 °C without shaking. Next, cells were centrifuged and cell pellets were washed with fresh BSK-H medium (1 mL) followed by resuspension in 500 μL of the same medium without antibiotics. Then, 50 μL of cell suspension was inoculated into 1 mL of fresh BSK-H medium, incubated at 33 °C for 20 days for subculture. Cell growth was assessed using SYBR Green I/PI assay and fluorescence microscopy, as described above. 

## 3. Results

### 3.1. Evaluating Activity of Essential Oils Against Stationary Phase B. Burgdorferi

**Figure 1:**
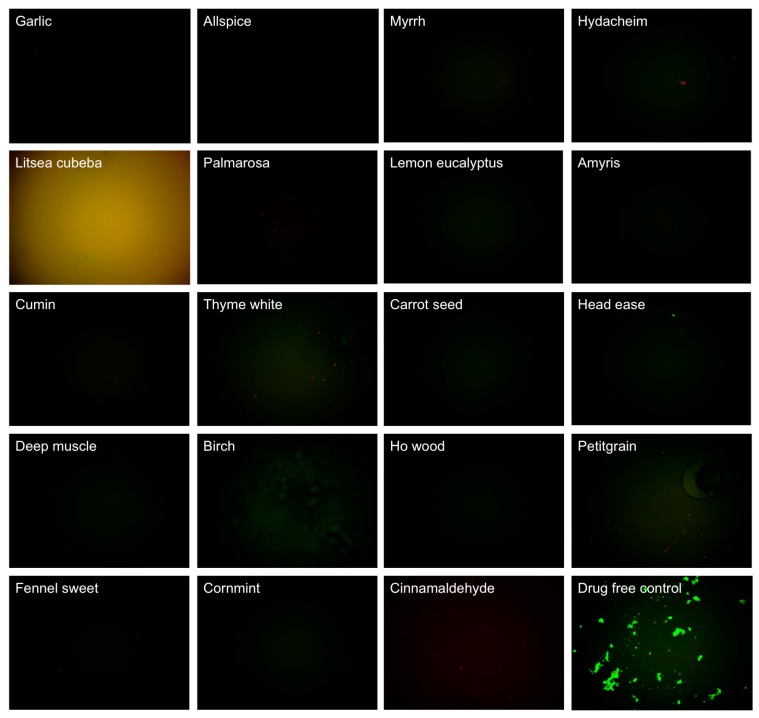
Effect of 0.2% essential oils on the viability of stationary phase *B. burgdorferi.* A 7-day old *B. burgdorferi* stationary phase culture was treated with 0.2% (*v/v*) essential oils for seven days followed by staining with SYBR Green I/PI viability assay and fluorescence microscopy.

**Figure 2:**
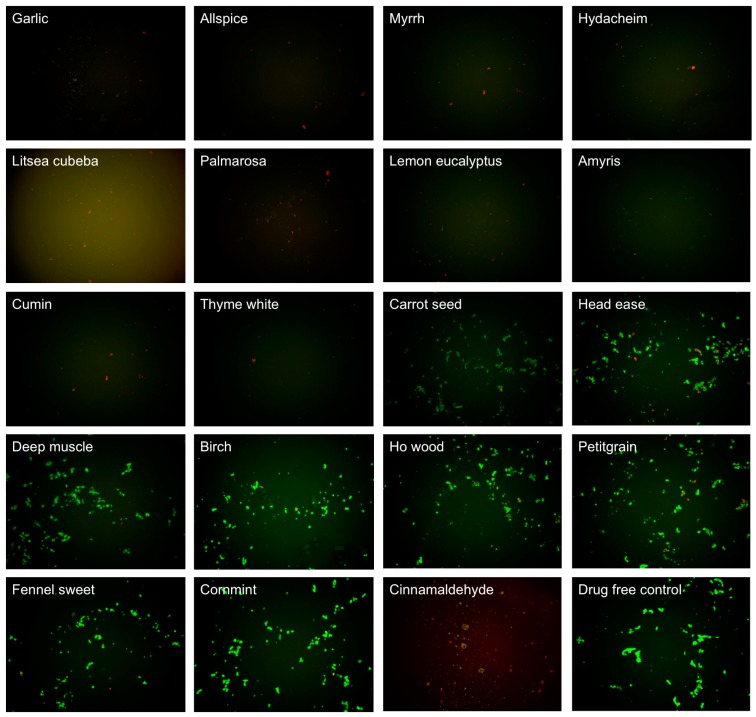
Effect of 0.1% essential oils on the viability of stationary phase *B. burgdorferi.* A seven-day old *B. burgdorferi* stationary phase culture was treated with 0.1% (*v/v*) essential oils for seven days followed by staining with SYBR Green I/PI viability assay and fluorescence microscopy.

In this study, we explored activity of another panel of 35 new essential oils together with control drugs against a seven-day old *B. burgdorferi* stationary phase culture in 96-well plates incubated for seven days. Our previous study discovered that cinnamon bark essential oil showed very strong activity against *B. burgdorferi* culture at stationary phase even at 0.05% concentration [18]. To identify the active components of cinnamon bark essential oil, we also added cinnamaldehyde (CA), the major ingredient of cinnamon bark, in this screen. Table 1 outlines the activity of the 35 essential oils and CA against *B. burgdorferi* culture at stationary phase. Although the *Litsea cubeba* essential oil showed too strong autofluorescence to determine its activity at 0.2% concentration, all the other essential oil candidates, except parsley seed, showed significantly stronger activity (*p* < 0.05) than the doxycycline control (Table 1) at 0.2% concentration with SYBR Green I/PI assay. Among them, 16 essential oils and CA at 0.2% concentration were found to have strong activity against *B. burgdorferi* culture at stationary phase as compared to the control antibiotics doxycycline, cefuroxime, and daptomycin (Table 1). As previously described [23], we calculated the ratio of residual live cells and dead cells of microscope images using Image Pro-Plus software, which could eliminate the autofluorescence of the background. Using fluorescence microscopy, we confirmed that, at 0.2% concentration, the 16 essential oils and CA could eradicate all live cells with only dead and aggregated cells left as shown in Table 1 and Figure 1. At concentration of 0.1%, 10 essential oils (garlic, allspice, cumin, palmarosa, myrrh, hydacheim, amyris, thyme white, *Litsea cubeba*, lemon eucalyptus), and CA still exhibited significant activity (*p* < 0.05) over the current clinically used doxycycline (Table 1; Figure 2). Among them, the most active essential oils were garlic, allspice, cumin, palmarosa, myrrh, and hydacheim because of their remarkable activity even at 0.1%, as shown by totally red (dead) cells with SYBR Green I/PI assay and fluorescence microscope tests (Figure 1). CA also showed very strong activity at 0.1% concentration. Although the plate reader data showed carrot seed and deep muscle essential oils had a significant activity (*p* < 0.05) compared with the doxycycline control, the microscope result did not confirm it due to high residual viability (60% and 68%, *p* > 0.05) (Table 1). For the other six essential oils (cornmint, fennel sweet, ho wood, birch, petitgrain, and head ease), which showed strong activity at 0.2% concentration, we did not find them to have higher activity than the doxycycline control at 0.1% concentration (Table 1, Figure 2). In addition, although essential oils of birch and *Litsea cubeba* have autofluorescence, which showed false high residual viability and interfered with the SYBR Green I/PI plate reader assay, they both exhibited strong activity against the stationary phase *B. burgdorferi,* as confirmed by SYBR Green I/PI fluorescence microscopy.

**Figure 3:**
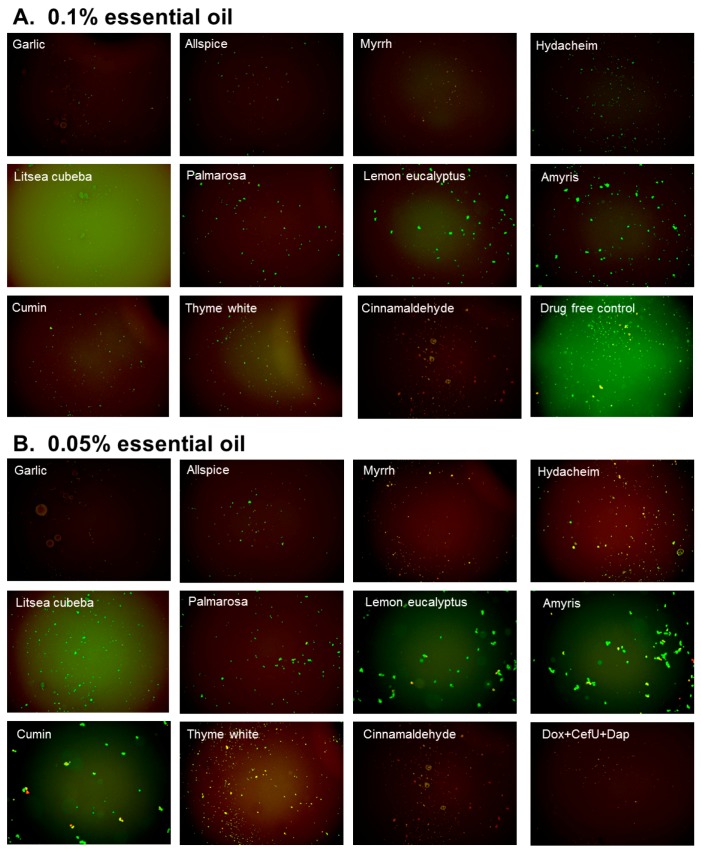
Effect of active essential oils on stationary phase *B. burgdorferi*. A 1 mL *B. burgdorferi* stationary phase culture (seven-day old) was treated with 0.1% (**A**) or 0.05% (**B**) essential oils (labeled on the image) in 1.5 mL Eppendorf tubes for 7 days followed by staining with SYBR Green I/PI viability assay and fluorescence microscopy.

The top 10 essential oils and CA (residual viability lower 60%) were chosen to evaluate their activities and explore their potential to eradicate *B. burgdorferi* cultures at stationary phase that harbor large numbers of persisters using lower essential oil concentrations (0.1% and 0.05%). We did the confirmation tests with 1 mL stationary phase *B. burgdorferi* in 1.5 mL Eppendorf tubes. At 0.1% concentration, the tube tests confirmed the active hits from the previous 96-well plate screen, although the activity of all essential oils decreased slightly in the tube tests when compared to the 96-well plate tests (Table 2, Figure 3). At a very low concentration of 0.05%, we noticed that garlic, allspice, palmarosa, and CA still exhibited strong activity against the stationary phase *B. burgdorferi*, approved by few residual green aggregated cells shown in Table 2 and Figure 3. Meanwhile, we also found CA showed strong activity against replicating *B. burgdorferi*, with an MIC of 0.02% (equal to 0.2 μg/mL).

### 3.2. Subculture Studies to Evaluate the Activity of Essential Oils Against Stationary Phase B. burgdorferi

**Figure 4:**
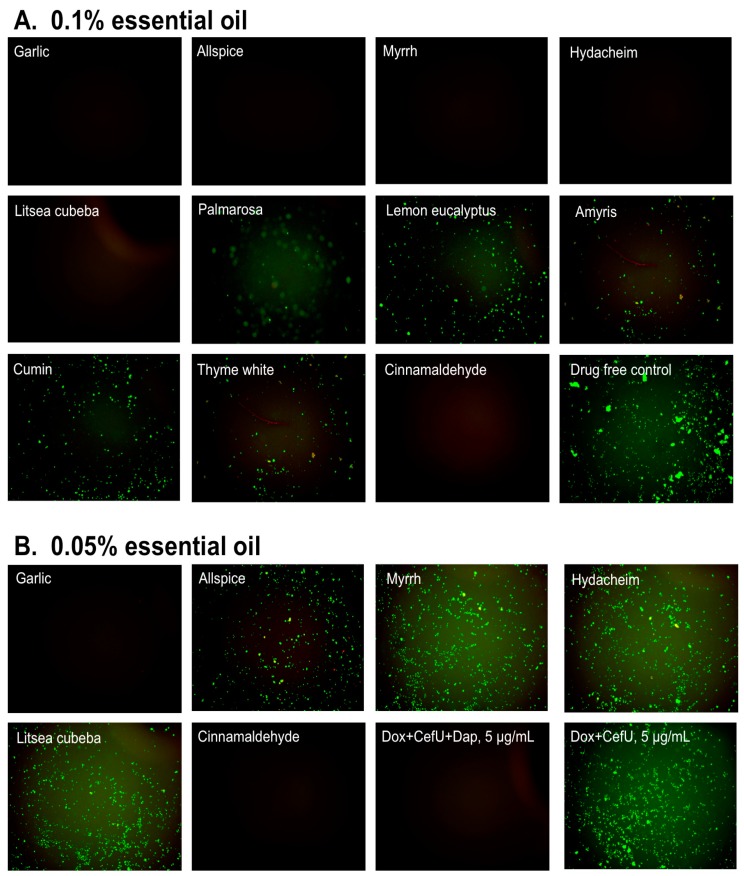
Subculture of *B. burgdorferi* after treatment with essential oils. A *B. burgdorferi* stationary phase culture (seven-day old) was treated with the indicated essential oils at 0.1% (**A**) or 0.05% (**B**) for seven days followed by washing and resuspension in fresh BSK-H medium and subculture for 21 days. The viability of the subculture was examined by SYBR Green I/PI stain and fluorescence microscopy.

To validate the capability of the essential oils in eradicating *B. burgdorferi* cells at stationary phase, we performed subculture studies by incubating essential oils treated cells in fresh BSK medium after the removal of the drugs with washing, as previously described [14]. We picked the top 10 active essential oils (garlic, allspice, myrrh, hydacheim, *Litsea cubeba*, palmarosa, lemon eucalyptus, amyris, cumin, and thyme white) to further confirm whether they could eradicate the stationary phase *B. burgdorferi* cells at 0.1% or 0.05% concentration by subculture experiments after the essential oil exposure (Table 2). At 0.1% concentration, we did not find any regrowth in samples of the top five hits, including garlic, allspice, myrrh, hydacheim, and *Litsea cubeba* (Table 2, Figure 4A). However, palmarosa, lemon eucalyptus, amyris, cumin and thyme white could not eradicate *B. burgdorferi* cells at stationary phase as many spirochetes were still visible after 21 days’ subculture (Figure 4A). The subculture study also confirmed the strong activity of CA by showing no growth of spirochete after treatment with 0.1% CA. At concentration of 0.05%, we did not observe spirochetal regrowth in the garlic essential oil treated samples that were subcultured for 21 days (Figure 4B), which indicates that garlic essential oil could completely kill all *B. burgdorferi* forms even at 0.05% concentration. On the other hand, the other four active essential oils (allspice, myrrh, hydacheim and *Litsea cubeba*) at a concentration of 0.05% could not sterilize the *B. burgdorferi* culture at stationary phase, since spirochetes were visible after 21 days subculture (Figure 4B). Similar to the previous subculture result of cinnamon bark essential oil [18], 0.05% cinnamaldehyde sterilized the *B. burgdorferi* stationary phase culture as shown by no regrowth after 21 days subculture (Figure 4B), indicating that the active component of cinnamon bark essential oil is attributable to cinnamaldehyde.

## 4. Discussion

We recently found that many essential oils have better activity against *B. burgdorferi* cells at stationary phase than the current clinically used antibiotics for treating Lyme disease [18]. Here, we screened another panel of 35 new essential oils using *B. burgdorferi* culture at stationary phase as a persister model [16]. Previously, we found that 23 essential oils had strong activity at 1% concentration, but only five of them showed good activity at a lower concentration of 0.25% [18]. To identify the essential oils that have activity against *B. burgdorferi* persisters at low concentrations, we performed the screen at 0.2% and 0.1% concentrations in this study. Some essential oils, such as *Litsea cubeba* oil, showed high autofluorescence after SYBR Green I/PI stain, which significantly interfered with the SYBR Green I/PI assay (Table 1, Figure 1). However, using lower concentration (0.1%) and fluorescence microscopy, we were able to verify the results from the SYBR Green I/PI assay and eliminate the problem of autofluorescence with some essential oils. Another limitation of the SYBR Green/PI assay is that not all cells turning red are dead, and further subculture studies are needed to verify whether the PI- stained red cells are indeed dead after drug exposure. In this study, we identified 18 essential oils (at 0.2% concentration) that are more active than 40 μM daptomycin (a persister drug control that could eradicate *B. burgdorferi* stationary phase cells), from which 10 essential oils stand out as having a remarkable activity even at 0.1% concentration (Table 1). Among them, garlic essential oil exhibited the best activity as shown by the lowest residual viability of *B. burgdorferi* at 0.1%. In the subsequent comparison studies, the garlic essential oil highlighted itself as showing a sterilizing activity even at a lower concentration of 0.05%, because no *Borrelia* cells grew up in the subculture study (Table 2). Garlic as a common spice has been used throughout history as an antimicrobial, and a variety of garlic supplements have been commercialized as tablets and capsules. The antibacterial activity of garlic was described by ancient Chinese, and in more recent times, by Louis Pasteur in 1858. Although allicin, an antibacterial compound from garlic, is shown to have antibacterial activity against multiple bacterial species [24,25], it has not been well studied on *B. burgdorferi*, especially the non-growing stationary phase organism, despite its anecdotal clinical use by some patients with Lyme disease (http://www.natural-homeremedies.com/blog/best-home-remedies-for-lyme-disease/; http://lymebook.com/blog/supplements/garlic-allimax-allimed-alli-c-allicin/). In this study, garlic essential oil was identified as the most potent candidate having activity against stationary phase *B. burgdorferi*, and its activity is equivalent to that of oregano and cinnamon bark essential oils, the two most active essential oils against *B. burgdorferi* we identified in our previous study [18]. 

Additionally, we found four other essential oils, allspice, myrrh, hydacheim, and *Litsea cubeba* that showed excellent activity against *B. burgdorferi* at the stationary phase, though the extracts or essential oils of these four plants were reported to possess antibacterial activity on other bacteria. Allspice is a commonly used flavoring agent in food processing and is known to have antibacterial activities on many organisms [26]. Myrrh as a traditional medicine has been used since ancient times. In modern times, myrrh is used as an antiseptic in topical and toothpaste [27]. It has been shown that some sesquiterpene components of myrrh, including furanodien-6-one, methoxyfuranoguai-9-en-8-one possess in vitro bactericidal, and fungicidal activity against multiple pathogenic bacteria, including *E. coli*, *S. aureus*, *P. aeruginosa*, and *C. albicans* [28], but these two most active compounds were not detected in our samples [19] (Table 3). Hydacheim essential oil is extracted from the flower of *Hedychium spicatum* plant which is commonly known as the ginger lily plant. The methanol extract of *H. spicatum* is reported to have antimicrobial activity against many bacteria, including *Shigella boydii*, *E. coli*, *S. aureus*, *P. aeruginosa,* and *K. pneumoniae* [29]. *Litsea cubeba* is also used in traditional Chinese medicine for a long time. Its essential oils from stem, alabastrum, leaf, flower, root, fruit parts are also reported to exhibit antibacterial activity on *B. subtilis*, *E. coli*, *E. faecalis*, *S. aureus*, *P. aeruginosa*, and *M. albicans* [30]. Based on these studies and application of allspice, myrrh, hydacheim, and *Litsea cubeba*, it would be of interest to develop effective regimens to fight against Lyme disease in the future. 

Although the active essential oils we identified have strong activity against stationary phase cells of *B. burgdorferi* in vitro, their activity in vivo is unknown at this time. In future studies, we will use GC-Mass Spectrometry to identify the active ingredients of the active essential oils and confirm their activity against growing and non-growing *B. burgdorferi*. Once we identify the active components of active essential oils drug combination studies can be performed to enhance activity against persister bacteria. In addition, we will study the mechanism of action of the active compounds in the near future. The pharmacokinetic (PK) profile of the active compounds in these active essential oils will be assessed and their effective dosage and toxicity will be determined in vivo. In our previous study, we found that the cinnamon bark essential oil showed excellent activity against stationary phase *B. burgdorferi* [18], here we found CA is an active component of cinnamon bark essential oil. CA could eradicate the stationary phase *B. burgdorferi* at 0.05% concentration as no regrowth occurred in subculture (Table 2). This indicates that CA possess similar activity against stationary phase *B. burgdorferi* as carvacrol, which is the one of the most active compounds against non-growing *B. burgdorferi* we identified from natural products [18]. Furthermore, CA is observed to be very active against growing *B. burgdorferi* cells with an MIC of 0.2 µg/mL. The antibacterial activity of CA was also reported on some other bacteria. The mechanism of the antibacterial activity of CA has been studied on different microorganisms, which suggests that its antibacterial action is mainly through interaction with the cell membrane [31]. CA as a common favoring agent in food processing is also used as food preservative to protect animal feeds and human food from pathogenic bacteria [31]. CA is considered as a safe compound for mammals, as the median lethal dose LD_50_ of CA is 1850 ± 37 mg/kg by oral administration in the acute toxicity study with oral administration rat model [32]. These findings suggest that CA could be a good drug candidate for further evaluation against *B. burgdorferi* in future studies. We also want to point out that the safety of using essential oils and their components needs more thorough research; for example, the intravenous toxicity of carvacrol is considerably higher than oral toxicity [33]. Thus, appropriate animal studies are necessary to confirm the safety and activity of CA and other active essential oils in animal models before human studies. 

This study used *B. burgdorferi* stationary phase cultures enriched in persisters as a persister model for essential oil screens. The reason we used this model is that studies with tuberculosis persister drug pyrazinamide (PZA), which is more active against stationary phase cells and persisters than against log phase growing cells and shortens the therapy [34,35], suggest that drugs active against stationary phase cells/persisters will be more effective at curing persistent infections than drugs active against growing cultures. This has been shown in the case of colistin as a persister drug for *E. coli* when being used together with quinolone or nitrofuran could more effectively eradicate urinary tract infection in mice [36]. Future studies are needed to determine if the essential oils active against non-growing stationary phase *B. burgdorferi* cultures enriched in persisters are more effective in eradicating persistent *B. burgdorferi* infections in animal models than the current Lyme antibiotics which are mainly active against growing *Borrelia*.

## 5. Conclusions

In summary, we identified some additional essential oils that have strong activity against stationary-phase cells of *B. burgdorferi.* The most active essential oils include garlic, allspice, myrrh, hydacheim, and *Litsea cubeba*. Among them, garlic oil could completely eradicate stationary phase *B. burgdorferi* with no regrowth at 0.05%, and the others could reach the same activity at 0.1%. Additionally, cinnamaldehyde is identified to be an active ingredient of cinnamon bark oil with very strong activity against *B. burgdorferi* stationary phase cells. Future studies will be carried out to identify the active components in the candidate essential oils, and to determine their in vitro activity alone or in combination with other active essential oils or antibiotics against *B. burgdorferi* sensu lato strains, including *B. burgdorferi, B. garinii* and *B. afzelii,* and assess their safety and efficacy against *B. burgdorferi* in animal models before human trials. 

## Figures and Tables

**Table 1:** Effect of essential oils on a seven-day old stationary phase *B. burgdorferi* ^a^.

Essential Oils and Control Drugs	Plant and Extracted Part	Residual Viability (%) after 0.2% EO or 40 μM Antibiotic Treatment			Residual Viability (%) after 0.1% EO Treatment		
		Plate Reader ^b^	Microscope ^c^	*p*-Value ^e^	Plate Reader ^b^	Microscope ^c^	*p*-Value ^e^
Doxycycline	--	73 ± 4	66 ± 2	1.000	--	--	--
Cefuroxime	--	58 ± 3	55 ± 3	0.0016	--	--	--
Daptomycin	--	28 ± 5	21 ± 1	0.0004	--	--	--
Cinnamaldehyde	--	26 ± 5	0	0.0002	54 ± 2	28	0.0016
Garlic	*Allium sativum* L. (syn. *Porrum sativum* (L.) Rchb.), bulbs	25 ± 4	0	0.0001	24 ± 6	18 ± 2	0.0002
Allspice	*Pimenta officinalis* Lindl., berries	21 ± 4	0	0.0001	30 ± 6	25 ± 3	0.0005
Myrrh	*Commiphora myrrha* (T. Nees) Engl. (syn. *Balsamea myrrha* (T. Nees) Oken), resin	32 ± 3	0	0.0001	35 ± 6	25 ± 2	0.0009
Hydacheim	*Hedychium spicatum* Thymus (syn. *Gandasulium spicatum* (Buch.-Ham. ex Sm.) Kuntze), flowers	34 ± 4	23 ± 2	0.0002	38 ± 7	26 ± 1	0.0017
*Litsea cubeba*	*Litsea cubeba* (Lour.) Pers. (syn. *Benzoin cubeba* (Lour.) Hatus., *Persea cubeba* (Lour.) Spreng.), fruits	98 ± 4	ND ^d^	--	77 ± 4	27 ± 3	(0.00004)
Palmarosa	*Cymbopogon martini* var. *motia* Bruno, grass	26 ± 5	0	0.0002	35 ± 5	29 ± 2	0.0004
Lemon eucalyptus	*Eucalyptus citriodora* Hook. (syn. *Corymbia citriodora* (Hook.) K.D. Hill & L.A.S. Johnson), leaves	35 ± 6	0	0.0006	39 ± 7	29 ± 4	0.0015
Amyris	*Amyris balsamifera* L. (syn. *Elemifera balsamifera* (L.) Kuntze), wood	32 ± 3	4 ± 2	0.0001	38 ± 5	29 ± 3	0.0006
Cumin	*Cuminum cyminum* L., seeds	31 ± 3	0	0.0001	31 ± 6	30 ± 1	0.0005
Thyme white	*Thymus vulgaris* L. (syn. *Origanum thymus* (L.) Kuntze), leaves	37 ± 2	26 ± 2	0.0001	36 ± 1	30 ± 2	0.0001
Carrot seed	*Daucus carota* L., seeds	38 ± 4	5 ± 3	0.0004	40 ± 3	60 ± 2	0.0003 (0.0705)
Head ease	Synergy blend	41 ± 3	25 ± 3	0.0003	74 ± 4	65 ± 1	0.8008
Deep muscle	Synergy blend	42 ± 4	3 ± 2	0.0004	56 ± 4	68 ± 4	0.0060 (0.3911)
Birch	*Betula lenta* L., bark	86 ± 5	22 ± 2	(0.00001)	91 ± 4	69 ± 2	--
Ho wood	*Cinnamomum camphora* (L.) J. Presl (syn. *Cinnamomum camphora* (L.) Nees & Eberm., *Cinnamomum camphora* (L.) Siebold), twigs and bark	36 ± 4	3 ± 2	0.0004	69 ± 5	70 ± 3	0.3078
Petitgrain	*Citrus aurantium* L, trees and leaves	38 ± 3	19 ± 2	0.0002	71 ± 4	70 ± 3	0.4743
Fennel sweet	*Foeniculum vulgare* Mill., seeds	40 ± 5	2 ± 1	0.0006	72 ± 3	75 ± 4	0.6235
Cornmint	*Mentha arvensis,* leaf	35 ± 5	0	0.0004	68 ± 4	85 ± 1	0.1359
Citrus blast	Synergy blend	51 ± 5	>70	0.0039	71 ± 5	>70	0.5865
Nutmeg	*Myristica fragrans* Houtt., seeds	43 ± 4	>70	0.0008	71 ± 4	>70	0.6533
Alive	Synergy blend	40 ± 4	>70	0.0004	71 ± 3	>70	0.5228
New beginning	Synergy blend	48 ± 4	>70	0.0013	75 ± 4	>70	0.5107
Happy	Synergy blend	47 ± 4	>70	0.0009	78 ± 2	>70	--
Meditation	Synergy blend	55 ± 4	>70	0.0041	79 ± 4	>70	--
Deep forest	Synergy blend	61 ± 1	>70	0.0039	79 ± 3	>70	--
Copaiba	*Copaifera officinalis* (Jacq.) L., balsm	51 ± 2	>70	0.0007	79 ± 2	>70	--
Balsam fir	*Abies balsamea* (L.) Mill. (syn. *Peuce balsamea* (L.) Rich.), needles	57 ± 5	>70	0.0124	80 ± 1	>70	--
Juniper Berry	*Juniperus communis* L., berries	56 ± 5	>70	0.0086	82 ± 3	>70	--
Camphor	*Cinnamomum camphora* (L.) J. Presl (syn. *Cinnamomum camphora* (L.) Nees & Eberm., *Cinnamomum camphora* (L.) Siebold), wood	58 ± 3	>70	0.0047	82 ± 3	>70	--
Vetiver	*Vetiveria zizanioides* (L.) Nash (syn. *Phalaris zizanioides* L.), root	41 ± 3	>70	0.0003	82 ± 5	>70	--
Fir needle	*Abies sibirica* Ledeb. (syn. *Pinus sibirica* (Ledeb.) Turcz.), needles	60 ± 3	>70	0.0109	83 ± 5	>70	--
Sleep tight	Synergy blend	57 ± 5	>70	0.0130	85 ± 6	>70	--
Turmeric	*Curcuma longa* L. (syn. *Kua domestica* (L.) Medik.), root	50 ± 2	>70	0.0007	93 ± 3	>70	--
Elemi	*Canarium luzonicum* (Blume) A. Gray (syn. *Pimela luzonica* Blume), resin	58 ± 3	>70	0.0059	95 ± 2	>70	--
Parsley seed	*Petroselinum sativum* Hoffm., seeds	64 ± 5	>70	0.0645	97 ± 3	>70	--

^a^ A seven-day old *B. burgdorferi* stationary phase culture was treated with essential oils or control drugs for 7 days. Bold type indicates the essential oils that had better or similar activity compared with 40 μM daptomycin used as the positive persister-drug control. ^b^ Residual viable (mean ± SD) *B. burgdorferi* was calculated according to the regression equation and ratios of Green/Red fluorescence obtained by SYBR Green I/PI assay [22]. ^c^ Residual viability (mean ± SD) calculated by fluorescence microscope measurements. ^d^ Autofluorescence of essential oil is too strong to be observed under fluorescence microscope. ^e^ *p*-value of the standard *t*-test for the 0.1% essential oil treated group versus doxycycline treated control group was calculated by data of the plate reader or microscope test (shown in the brackets). The essential oil groups with higher residual viability than control group were not included in the standard *t*-test.

**Table 2:** Comparison of top 10 essential oil activities against stationary phase *B. burgdorferi* with 0.1% and 0.05% (*v/v*) treatment and subculture ^a^.

Essential Oil Treatment	Residual Viability after 0.1% Essential Oil Treatment		Residual Viability after 0.05% Essential Oil Treatment	
	Treatment ^b^	Subculture ^c^	Treatment ^b^	Subculture ^c^
Drug free control	93%	+	93%	+
Daptomycin+Doxycycline+Cefuroxime ^d^	18%^d^	− ^d^	N/A	N/A
Garlic	30%	−	33%	−
Allspice	34%	−	48%	+
Myrrh	42%	−	41%	+
Hydacheim	44%	−	61%	+
*Litsea cubeba*	68%	−	69%	+
Palmarosa	39%	+	67%	+
Lemon eucalyptus	46%	+	79%	+
Amyris	48%	+	71%	+
Cumin	42%	+	60%	+
Thyme white	40%	+	76%	+
Cinnamaldehyde	34%	−	56%	−

^a^ A 7-day old stationary phase *B. burgdorferi* was treated with 0.05% or 0.1% essential oils for seven days when the viability of the residual organisms was assessed by subculture. ^b^ Residual viable percentage of *B. burgdorferi* was calculated according to the regression equation and ratio of Green/Red fluorescence obtained by SYBR Green I/PI assay as described [22]. Viabilities are the average of three replicates. ^c^ “+” indicates growth in subculture; “−” indicates no growth in subculture. ^d^ Activity was tested with 5 μg/mL of each antibiotic in combination.

**Table 3:** The top five major compositions of the three most active essential oils.

Essential Oil	Components	Content ^a^
Garlic *Allium sativum* bulbs	Diallyl disulfide	19%
	(E)-1-Allyl-2-(prop-1-en-1-yl) disulfane	15%
	Disulfide, methyl 2-propenyl	6%
	2-Vinyl-4H-1,3-dithiine	6%
	(E)-methyl 1-propenyl sulfide	4%
Allspice *Pimenta officinalis* berries	Eugenol	82%
	β-Caryophyllene	6%
	Methyleugenol	5%
	α-Humulene	1%
	α-Selinene	0.47%
Myrrh *Commiphora myrrha* resin	Curzerene	38%
	Furanoeudesma-1,3-diene	24%
	β-Elemene	7%
	Lindestrene	7%
	E-Elemene	3%

^a^ The content of components were calculated according to the GC-MS analysis in our lab (Garlic essential oil) and PhytoChemia Laboratories reports on Plant Guru company website (Allspice and Myrrh) [19].
